# Sleep Health and Falls Risk for Older Adults Living in Residential Aged Care and in Community Dwelling Settings: A Longitudinal Observation Study

**DOI:** 10.1177/00469580241306274

**Published:** 2024-12-17

**Authors:** Samantha Fien, Natasha L. Bennett, Patrick J. Owen, Stephanie J. Alley, Corneel Vandelanotte, Madeline Sprajcer, Kim Waters, Steven T. Moore, Justin W.L. Keogh, Grace E. Vincent

**Affiliations:** 1 Central Queensland University, Mackay, QLD, Australia; 2 Eastern Health Emergency Medicine Program, Melbourne, VIC, Australia; 3 Eastern Health Clinical School, Monash University, Melbourne, VIC, Australia; 4 Appleton Institute, Central Queensland University, Rockhampton, QLD, Australia; 5 Appleton Institute, Central Queensland University, Wayville, SA, Australia; 6 Central Queensland University, Rockhampton, QLD, Australia; 7 Bond University, Gold Coast, Australia; 8 AUT University, Auckland, New Zealand; 9 Kasturba Medical College, Mangalore, Karnataka, India

**Keywords:** sleep, falls, falls risk, residential aged care, community-dwelling

## Abstract

This study explored measures of subjective and objective sleep health and the association with fall occurrence and falls risk for older adults. A longitudinal observational study was conducted with participants in residential aged care (n = 36) and community dwelling (n = 35) settings. At baseline, objective sleep data involved wearing wrist worn accelerometers and measuring falls risk by walking using the Quantitative timed up and go (QTUG) of a simple, cognitive, and motor task. Subjective sleep data was collected by completing sleep diaries using the Karolinska Sleepiness Scale and sleep quality scale, respectively. Longitudinal falls data were collected at baseline, 3, 6, and 9 months. Falls risk was calculated via QTUG. Responses to a fall questionnaire were used to quantify fall occurrence. Independent samples *t*-test examined differences in objective and subjective sleep variables between settings. Logistic regression explored whether objective or subjective sleep variables could predict an overall fall occurrence. Linear regression determined if a particular sleep variable could predict an overall falls risk. Multiple regression determined if sleep variables could predict falls risk. No significant differences were found between residential and community-dwelling adults in subjective or objective sleep measures. Logistic regression showed no significant associations between most sleep variables and falls risk, except for average awakening length, where each additional minute was associated with a 1.8% increase in fall likelihood (OR = 1.02, 95% CI [1.00-1.03], *P* = .037). Conversely, higher awakening frequency was associated with reduced falls risk in the simple timed up-and-go task (*R*² = .21, β = −.69, *P* = .009, 95% CI [−1.20 to −0.18]). Findings suggest no significant differences in sleep health or falls risk between residential and community-dwelling older adults, though specific sleep disruptions showed minor associations with falls risk.

## Introduction

Sleep is fundamental for human health and wellbeing.^1,2^ Across the lifespan, sleep changes substantially, with sleep deterioration common in older adults ≥65 years.^2^ Older adults often experience poor sleep due to insufficient sleep duration and/or regular sleep disruptions, that is, sleep fragmentation.^3^ Poor sleep can adversely impact cognitive and physiological functioning as well as the cardiovascular system, immune system, and hormonal system.^1,3^ Poor sleep can negatively impact motor functions that play a critical role in balance, coordination, and movement, which may increase the risk of falls in older adults.^1,3^

Falls are a leading cause of mortality in older adults.^2^ Globally, 26.5% of older adults aged ≥65 years had a fall-related injury over the last 12 months, requiring medical attention and/or hospitalization.^4^ Fall-related hospitalizations are five times more likely to occur than any other injury in older adults.^4^ In Australia, falls are the most common hospitalized injury (77%) and injury-related deaths (71%) for older adults.^5^ Fall related injuries cost the Australian healthcare system $4.7 billion per annum, with patients spending on average 9.5 days in hospital.^5,6^ From a financial and burden of disease perspective, it is critical to mitigate the risk of falls in an aging population.^4^

In Australia, the majority of older adults reside in community dwelling settings with 99% aged 65 to 84 years and 75% aged ≥85 years, that is, personal home, unit, apartment, public housing, or retirement villages.^7^ Older adults that reside in residential aged care are commonly aged ≥85 years.^7^ Residential aged care is defined as a facility providing accommodation and ongoing full-time care for individuals who can no longer remain independent in their own home.^8^ It is recommended that older adults have a sleep duration of 7 to 9 h per night.^9^ A 2021 survey found that older adults aged ≥65 years sleep an average of 7 h per night; 20% of whom report poor sleep quality and sleep disruptions.^10^

Older adults often experience sleep disruptions, including a greater proportion of time spent in lighter sleep stages of rapid eye movement and Stage 1, resulting in a greater likelihood of being woken and less time spent in deeper stages of sleep in Stage 2 and Stage 3.^1,11^ Deeper stages of sleep are important for restoration to improve brain health and function.^1,11^ Additionally, older adults living in residential aged care have reported additional environmental contributors to sleep disturbances^12^ such as inappropriate light and noise exposure, routine checks by staff, and disturbances from other residents.^11-13^ While differences in sleep stage duration between community-dwelling and residential aged care adults have not been previously investigated, older adults in residential aged care settings can feel unrested upon awakening and are inclined to nap throughout the day.^1,3,12^ There is evidence to suggest that short sleep duration and daytime sleepiness may be associated with an increased risk of falls amongst older adults.^14^

Impaired sleep can have multiple adverse consequences, one of which may be an increased risk of falls.^2^ One study found that older adults residing in residential aged care and community dwelling settings, who napped >30 min during the day or slept at night were at a higher risk of having multiple falls compared to older adults who did not nap or slept >6 h at night (OR = 3.10, 95% CI [1.30-7.42] and OR = 3.13, 95% CI [1.33-7.37], respectively).^13^ It was also reported that napping occurred more frequently in residential aged care compared to community dwelling settings, after controlling for age (*P* *=* .005).^13^ Similarly, another study found that older adults in residential aged care reported poorer sleep quality (95.8%) compared to community dwelling adults (76%).^15^ However, this evidence is inconsistent. For example, a study by St George et al^13^ found that night-time sleep disruptions were similar between older adults aged 62 to 95 years, regardless of residing in residential aged care or in community dwelling settings. Furthermore, sleep quality has been found to impact quality of life in older adults, regardless of their residing environment.^15^ Given these conflicting outcomes, it is imperative that sleep and falls continue to be investigated in both residential and community dwelling older adults.^15,16^

There is limited research that examines which sleep measures, both objective and subjective, best predict the risk of a fall in older adults.^11,17^ Studies have found that short sleep duration, sleepiness during the day, and napping are all associated with increasing the risk of a fall.^14,18^ Other objective sleep parameters such as sleep efficiency, latency before sleep onset, and sleep fragmentation have not yet been explored as predictors for falls risk.^19^ While sleep diaries are a valuable tool to collect subjective data; relying solely on participant responses can be prone to inaccuracies.^20^ Thus, it is important to combine both objective and subjective measures to increase accuracy of results as self-reported data can be prone to recall bias, for example; overestimating or underestimating the duration of sleep and number of awakenings.^14,21^ Given the limited research on sleep and falls in the older adults, this longitudinal observational project aimed to explore sleep health by investigating objective (using validated wrist-worn accelerometers) and subjective (perception of sleep quality and quantity) sleep domains, assessing whether these sleep measures are associated with the risk of falls among older adults in residential aged care and community-dwelling settings. The primary objective of this study was to evaluate the relationship between sleep and falls, with the following hypotheses: (1) objective and subjective measures of sleep health will be poorer in residential aged care adults than in community-dwelling adults; and (2) both objective and subjective sleep measures will be associated with the risk of falls in both settings.

## Methods

### Design

This longitudinal observational study was conducted in Queensland, Australia whereby data collection of sleep and falls occurred at four time points over 9 months in 2021. This study has followed cohort STROBE guideline^22^ (Supplemental File 1).

### Recruitment and Participants

**Figure 1.**
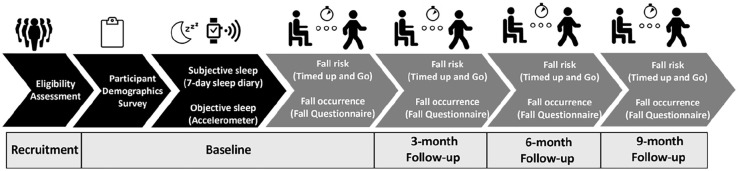
Sleep health and falls risk protocol.

An overview of recruitment and study protocol are presented in Figure 1.

Older adults were recruited from two Australian residential aged cares, with residential aged care staff, for example, physiotherapist or nurse identifying eligible residents. Community dwelling older adults were recruited through advertisements located at an exercise-based program via information sessions and posters on one campus of the university campuses located in Australia and within the community living environment surrounding the residential aged cares. To be eligible, participants had to be aged over 60 years,^23^ had to be able to provide informed consent and follow instructions - cognition deemed acceptable by the staff at the residential aged cares using their respective cognition tools and by the Mini-Mental State Examination^24^ assessment for community-dwelling older adults, and either reside in a residential aged care facility for more than 3 weeks or be living independently in the community. Exclusion criteria included having a life expectancy of less than 6 months, being unable to communicate or follow instructions given by the research staff, for example, cognitively impaired, or if participants engaged in behaviors, for example, aggressive, and agitated behaviors which either endangered themselves or research staff. All eligible participants were given an information sheet about the study and those that were willing to participate provided informed written consent before commencing the study. The accepted sample size for a longitudinal observation study is 30 participants or greater.^25,26^ Sample size calculations were performed in *G Power*.^27^ To detect statistically significant results with a power of .95 with an alpha level at .05 and a minimum of 30 participants in each setting (residential aged care and community dwelling) were required.^28^ Therefore, for this study, the sample consisted of participants from two residential aged care (n = 36) and community dwelling (n = 35).

## Measures

### Demographic Questions

Demographic information that was collected included age, location (residential aged care or community dwelling), gender, primary language spoken, and walking ability (no walking aid required, wheeled walker, or walking stick).

### Objective Sleep

To collect objective sleep data, participants were given a wrist accelerometer ActiGraph GT9X-Link (ActiGraph, Florida, United States of America), and were instructed to wear on the non-dominant wrist for 24 h per day, over a 7-day period. Wrist worn accelerometers captured movement through a monitor worn on the non-dominant wrist.^29,30^ Generally, an in-built accelerometer will detect movement at 1-min epochs (intervals).^30^ Previous studies have shown that actigraphy is valid for estimating sleep quality and sleep duration across all ages including older adults.^11,17^ Studies that have used objective measures, for example, actigraphy to measure sleep in older adults^11,17,31,32^ suggest that the following key variables be included for a comprehensive assessment of sleep; time in bed (minutes), total sleep time after sleep onset (min), number of awakenings, and minutes awake after sleep onset.^30^ It is recommended that actigraphy be used in conjunction with subjective measures, for accuracy and robustness.^17^ Self-reported sleep onset time and wake time provided in sleep diaries were used as guidelines to distinguish sedentary time from sleep periods from the wrist accelerometers, as per actigraphy protocol.^33^ The validated objective sleep parameters^34,35^ that were collected can be found in Supplemental File 2.

#### Falls risk

Falls risk data were collected using quantitative Kinesis QTUG™ software using an algorithm that included the QTUG task for simple, cognitive, and motor and the falls history questionnaire.^36^ Scores were calculated using participants’ QTUG test time compared against population data for age and gender, to estimate a falls risk score under 3 different conditions:

(1) a simple task that required the participant to stand from a chair, walk 3 m, turn, and walk 3 m back to the chair,
(2) a cognitive task that required participants to count backwards from 100 × 7, whilst performing the simple task, and
(3) a motor task that required participants to carry a glass of water while performing the simple task.

The falls risk score was estimated by the length of time taken to complete the task, that is, simple task, cognitive task, and motor task. A higher score indicated a higher risk of a fall.^36^ The falls risk score was used as an outcome to predict future risk of falls.

### Number of Falls

Participants were asked to complete a questionnaire relating to history of falls. There were 6 “yes” or “no” falls related questions. Falls history was whether the participant had fell in the last 12 months prior to baseline, and then if the participant had fallen in the 3 months at the follow up point at 3, 6, and 9 months. Furthermore, falls history was used as an outcome to explore if a sleep parameter could predict a fall, that is, yes = a fall occurred, no = no fall occurred.

### Falls Risk

Falls risk data were collected from the 3 QTUG activities of simple, cognitive, and motor tasks at baseline, 3, 6, and 9 months and were averaged for ease of analysis. The falls risk for each category was used in analyses in a predictor model to estimate if a sleep parameter could predict a falls risk. The falls risk was calculated through an algorithm within Kinesis using the QTUG activities and answers from the falls questionnaire to provide a falls risk for each participant. The higher the percentage, that is, closest to 100%, indicated a higher risk of falling.^36^

### Preparation of Objective Sleep Variables

Objective sleep data were analyzed using Actilife 6 Software (Actigraph, FL, USA). The Cole-Kripke algorithm as recommended by the Actilife 6 User’s Manual for adults aged 35 years and above and utilized in other studies^30,33^ was selected. The Actilife 6 Software, when paired with ActiGraph devices, includes an auto-detect sleep function that leverages accelerometer data to identify periods of sleep and wakefulness. This function typically assesses movement data, and classifies intervals of low activity as sleep periods. The auto-detect feature can improve data accuracy by automatically determining sleep onset and wake times without manual inputs, which helps reduce user or researcher error in sleep diaries. This approach is particularly useful in studies where objective measurement of sleep duration and fragmentation is important, as it provides standardized assessments of sleep across participants, ensuring consistency in data analysis and interpretation. The sleep diary entries given (self-reported sleep onset times and times they woke up) were utilized as guidelines to add sleep periods into the software.^30^ A total of 10 participants had not completed their sleep diary and the autodetect sleep period function was used. All data was averaged across the number of days the accelerometer was worn minimum of 2 days and maximum of 7 days. Additionally, 35 participants did not wear the accelerometer.

### Subjective Sleep

To collect subjective sleep data, participants were given a paper sleep diary and were asked to complete entries across the same 7-day period. After the 7-day period at baseline was completed, the research team collected the wrist accelerometers and paper sleep diaries from participants.

A sleep diary contained the following domains: time they fell asleep, time of wake up, number of times woken during the night, total time awake in minutes, number of hours asleep, score how sleepy they felt when they woke up via the 1 to 9 Karolinska Sleepiness Scale (KSS),^37^ and a sleep quality score on a 1 to 5 Likert Scale.^38^ All sleep diary domains were used as outcomes or predictors for statistical analysis.

### Karolinska Sleepiness Scale (KSS)

The KSS^37^ was used as a measure of sleepiness in the sleep diary. Participants used this scale to report their level of sleepiness upon waking each morning. The scale is scored as; 1 = extremely alert, 2 = very alert, 3 = alert, 4 = rather alert, 5 = neither alert nor sleepy, 6 = some signs of feeling sleepy, 7 = sleepy, no effort to keep awake, 8 = sleepy, some effort to keep awake, 9 = very sleepy, much effort to keep awake and fighting sleep.^37^ The KSS is considered to have good validity and has been widely utilized in studies that include shift work, sleep deprivation, and sleepiness during driving.^37^ The KSS has good overall inter-rater reliability with a Kappa value of *K* = 0.73.^37^ Usually, Kappa values between *K* = 0.61 to 0.80 are considered good agreement and *K* ≥ 0.81 are very good agreement.^37^

### Sleep Quality

A 1 to 5 Likert Scale provided insight to what level the person believed their sleep quality was; 1 = very well, 2 = well, 3 = average, 4 = poor, 5 = very poor.^39,40^

#### Falls questionnaire

Participants were asked to complete the falls questionnaire included within the software of the Kinesis device,^36^ which was included in the algorithm above to calculate falls risk. The falls questionnaire was conducted at baseline and repeated for follow up at 3, 6, and 9 months. Questions included, but not limited to on the topics of medication, walking/mobility, blood pressure, vision, previous falls.

### Preparation of Subjective Sleep Variables

For ease of analysis, each subjective outcome was averaged across the number of days the participant had made an entry with a minimum of 2 days and a maximum of 7 days. Additionally, some participants (n = 23) did not complete the sleep diary and all missing data were left blank.

## Statistical Analyses

All data were analyzed through Statistical Package for Social Sciences (SPSS) 29.0 Software (International Business Machines Corporation., Armonk, New York, USA). Statistical significance was set at an α of .05.

### Preliminary Analyses

A preliminary analysis was conducted on the categorical and continuous variables, using frequency and descriptive statistics, respectively. For continuous variables, the means, standard deviations are presented. The Shapiro-Wilk test was used to assess normality within the data and the Levene test was used to assess equality of variance.^41^

### Testing Hypotheses

**Hypothesis 1: Objective and subjective measures of sleep health will be poorer in residential aged care adults than in community dwelling adults.**

To test this hypothesis, an independent samples *t*-test was conducted to compare the means of each subjective and objective sleep variable between residential aged care settings and community dwelling settings. It was noted here that some variables violated assumptions of homogeneity of variance and normally distributed residuals, therefore the Mann-Whitney *U* Test was used for a sensitivity analysis.

**Hypothesis 2: Objective measures of sleep health will predict the risk of falls compared to subjective measures of sleep health.**

First, logistic regression was conducted to explore which sleep domain, all objective and subjective variables, could predict a fall (yes, no). Odds ratio output was used to explain the odds of experiencing a fall and whether this was statistically significant. Second, a linear regression model was conducted to explore relationships between each sleep variable and falls risk from simple, cognitive, and motor QTUG tests. Third, a multiple regression analysis was performed to determine if 3 predetermined objective sleep variables could predict the occurrence of falls. The variables included total sleep time, the number of awakenings, and the average length of each awakening. This analysis was conducted at 4 different time points: baseline, 3, 6, and 9 months. The selection of these specific sleep variables was based on previous research indicating that objective measures of sleep, such as total sleep time and sleep disturbances, tend to be more reliable than subjective measures, particularly in older adult populations.^12,13^ The multiple regression model was replicated to explore prediction of falls risk.

## Results

### Demographics

The demographic characteristics for participants in each group are summarized in Table 1. The sample consisted of residential aged care participants (n = 36) and community dwelling participants (n = 35). The full sample (N = 71) was predominantly female (74.6%). The mean age of participants in residential aged care was 85.7 ± 8.2 years, while the average age of community dwelling participants was 78.2 ± 5.7 years, see Table 1.

**Table 1.** Demographic Characteristics of Participants at Baseline.

Demographic Characteristics	Residential care		Community dwelling		Total	
	n	%	n	%	n	%
n	36	50.7	35	49.3	71	100.0
Gender						
Female	25	69.4	28	80.0	53	74.6
Male	11	30.6	7	20.0	18	25.4
Age group (years)						
60-69	0	0.0	3	8.6	3	4.2
70-79	9	25.0	17	48.6	26	36.6
80-89	14	38.9	15	42.8	29	40.9
90-99	13	36.1	0	0.0	13	18.3
Language						
English	36	50.7	35	49.3	71	100.0
Walking ability						
No walking aid required	6	16.7	33	94.3	39	54.9
Wheeled walker	29	80.5	2	5.7	31	43.7
Walking stick	1	2.8	0	0.0	1	1.4

*Note.* n = number of participants per group; % = the percentage of participants per group.

### Hypotheses Testing

**Hypothesis 1: No significant differences were found between objective and subjective measures of sleep health in residential aged care adults and in community dwelling adults.**

All subjective and objective variables averaged across 7 days are summarized in Table 2. On average (mean ± standard deviation), participants across the two groups reported feeling alert to very alert (2.8 ± 1.2 in residential aged care and 2.7 ± 1.2 in community dwelling settings) after sleeping and reported their sleep quality as average to well (2.7 ± 1.3 in residential aged care and 2.7 ± 1.2 in community dwelling settings). Participants reported their total sleep time was on average 7 h (7.2 ± 2.4 h in residential aged care settings and 6.9 ± 1.4 h in community dwelling settings). However, objective data indicated that total sleep time was less than subjectively reported (6.8 ± 2.2 h in residential aged care settings and 6.6 ± 1.7 h in community dwelling settings). There were no significant differences found between settings for all subjective and objective variables.

**Hypothesis 2: No significant differences were found in the objective and subjective measures of sleep health in predicting the risk of falls.**

**Table 2.** Subjective and Objective Sleep Variables Averaged Across 7-Days.

	Residential care	Community dwelling	*t*-Value (df)	*P-*value
Sleep Variables	*M ±* SD	*M ±* SD		
Subjective sleep variables (n = 45)				
Number awakenings	1.9 ± 1.4	2.2 ± 1.6	*t* (46) = −0.961	.342
Average awakening length (minutes)	47.2 ± 54.0	49.0 ± 58.7	*t* (46) = −0.518	.607
Sleepiness pre-sleep (1-9)	4.6 ± 1.6	4.0 ± 1.6	*t* (43) = 1.794	.080
Sleepiness post-sleep (1-9)	2.8 ± 1.2	2.7 ± 1.2	*t* (43) = 0.607	.547
Sleep quality (1-5)	2.7 ± 1.3	2.7 ± 1.2	*t* (45) = 0.259	.797
Total sleep time (h)	7.2 ± 2.4	6.9 ± 1.4	*t* (40) = 0.667	.509
Total time in bed (h)	7.9 ± 2.1	7.8 ± 1.3	*t* (46) = 0.496	.622
Objective sleep variables (n = 31)				
Latency (min)	6.1 ± 16.8	2.8 ± 5.6	*t* (32) = 0.980	.335
Sleep efficiency (%)	84.0 ± 14.7	85.6 ± 10.4	*t* (32) = −0.115	.910
Total time in bed (h)	8.1 ± 2.2	7.7 ± 1.7	*t* (32) = 1.052	.301
Total sleep time (h)	6.8 ± 2.2	6.6 ± 1.7	*t* (32) = 0.878	.386
Wake after sleep onset (min)	69.7 ± 61.2	61.9 ± 40.3	*t* (32) = 0.437	.665
Number awakenings	12.0 ± 8.7	15.0 ± 6.4	*t* (32) = −1.506	.142
Average awakening length (min)	6.1 ± 5.0	4.1 ± 1.7	*t* (32) = 1.595	.121
Movement index (%)	23.4 ± 15.4	17.1 ± 7.6	*t* (32) = 1.289	.207
Fragmentation index (%)	11.2 ± 12.0	11.1 ± 9.3	*t* (32) = −0.008	.993
Sleep fragmentation index (%)	34.6 ± 24.1	28.2 ± 13.7	*t* (32) = 0.829	.413

*Note. M* = mean parameter values for each variable; n = number of participants per group; SD = standard deviation in relation to the mean; *t*-value (df) = the *t* stastistic given from independent *t*-tests calculated with the degrees of freedom; min = minutes; h = hours; % = the percentage; *P*-value = statistical significance set at <.05.

The frequency of falls reported by residential aged care and community dwelling adults are summarized in Table 3. The majority of participants from each group reported having no falls across each time point.

**Table 3.** Frequency of Falls Reported by Participants at Each Timepoint.

Time point	Numbers of falls	Residential care fall occurrence	Community dwelling fall occurrence
Baseline (n = 68)			
	0	18	19
	1	6	10
	>2	9	6
3 months (n = 50)			
	0	21	18
	1	3	3
	>2	4	0
6 months (n = 31)			
	0	17	11
	1	0	3
	>2	0	0
9 months (n = 31)			
	0	12	13
	1	3	1
	>2	1	1

*Note.* n = number of participants per group.

Results from the logistic regression model at the baseline time point are shown in Table 4. This table presents if any sleep variables were able to predict the likelihood of a fall from the falls questionnaire at baseline. Following this analysis, this logistic regression model was replicated to explore if any sleep variables could predict a fall from the 3-month questionnaire, 6-month questionnaire, and 9-month questionnaire. There were no significant associations between sleep parameters and the likelihood of a fall; except for each minute awake (subjective sleep variable), the odds of experiencing a fall increased by approximately 1.8%, OR = 1.02, 95% CI [1.00-1.03], *P* = .037. Further, for each additional awakening (subjective sleep variable), the odds of experiencing a fall increased by approximately 65.3%, OR = 1.65, 95% CI [0.99-2.75], *P* = .053. Although this association was approaching statistical significance, it is just above the conventional 0.05 threshold.

**Table 4.** Logistic Regression Model to Predict a Fall at Baseline.

Predictors	Odds ratio	Standard error	*z-*Value	*P-*value	95% confidence interval (lower-upper)
Subjective sleep variables					
Number awakenings	1.652	0.428	1.94	.053	0.993-2.748
Average awakening length (min)	1.017	0.008	2.09	.037	1.001-1.034
Sleepiness pre-sleep (1-9)	1.083	0.253	0.34	.731	0.685-1.714
Sleepiness post-sleep (1-9)	1.762	0.663	1.50	.132	0.842-3.686
Sleep quality (1-5)	1.010	0.326	0.03	.974	0.536-1.904
Total sleep time (h)	0.963	0.185	−0.19	.846	0.660-1.404
Total time in bed (h)	1.195	0.271	0.78	.433	0.765-1.865
Objective sleep variables					
Latency (min)	0.965	0.051	−0.66	.510	0.870-1.071
Sleep efficiency (%)	0.999	0.040	−0.02	.982	0.922-1.081
Total time in bed (h)	1.000	0.003	0.21	.837	0.993-1.008
Total sleep time (h)	1.001	0.003	0.36	.722	0.993-1.008
Wake after sleep onset (min)	0.997	0.010	−0.23	.815	0.977-1.018
Number awakenings	1.002	0.063	0.03	.975	0.885-1.133

*Note.* Odds ratio explains the odds of experiencing a fall, *P*-value = statistical significance set at <.05. min = minutes; h = hours; % = the percentage.

Following the logistic regression analyses, a linear regression model was run to explore potential relationships between each subjective and objective sleep domain and falls risk (from simple, cognitive, and motor QTUG tests). Notably, additional awakenings (objective) throughout the sleep period, predicted a decreased falls risk during the simple task, *R*^2^ = .21, *F*(1,29) = 7.75, β = −0.69, *P* = .009, 95% CI [−1.20 to −0.18]. Similarly, additional awakenings (objective), predicted a decreased falls risk during the cognitive task, *R*^2^ = .13, *F*(1,29) = 4.52, β = −.50, *P* = .042, 95% CI [−0.98 to −0.02]. Therefore, a greater number of awakenings throughout the night was associated with a decrease in falls risk. There were no other significant associations.

Finally, the findings from the multiple regression model revealed no significant associations were found (refer to Supplemental File 3). The multiple regression model was replicated to explore if the same objective sleep variables (total sleep time, number awakenings, and average awakening length) could predict a higher falls risk. It was found that additional awakenings (objective) throughout the sleep period, predicted a decrease in the falls risk during the simple task, *R*^2^ = .24, *F*(3,27) = 2.90, β = −.64, *P* = .022, 95% CI [−1.17 to −0.10]. This indicated that if a participant awoke more during the night, was associated with a lower falls risk score. However, this finding did not remain statistically significant when predicting a falls risk during the motor task, *R*^2^ = .22, *F*(3,27) = 2.48, β = −.53, *P* = .064, 95% CI [−1.10 to 0.03], and during the cognitive task, *R*^2^ = .17, *F*(3,27) = 1.81, β = −.45, *P* = .085, 95% CI [−0.96 to −0.06].

## Discussion

This study examined the association between sleep and the risk of falls in older adults, both in residential aged care and community living environments. Our comparative analysis revealed no statistically significant differences in either objective or subjective sleep measures between these 2 settings. Additionally, our investigation into how various sleep measures might predict fall occurrence and falls risk yielded mostly statistically insignificant results. However, three notable exceptions were found: each minute spent awake (subjectively reported) increased the odds of experiencing a fall by 1.8%, for each additional awakening subjectively reported increased the odds of experiencing a fall by 65.3%, and a higher number of objectively measured awakenings during the night was consistently associated with a reduced risk of falls. This inverse relationship was observed in both the cognitive and simple QTUG.

Despite potential variations in sleep between residential aged care and community dwelling older adults, data revealed no notable differences in either subjective or objective sleep measures. This is in contrast with previous research, where participants in residential aged care settings were susceptible to more frequent sleep disturbances and poorer quality sleep when compared to community dwelling older adults.^11,12,15^ In the current study, we found that a substantial proportion of participants, including 47.6% from residential aged care and 53.3% from community dwelling environments, objectively slept less than the recommended 7 to 8 h for older adults as suggested by the National Sleep Foundation.^9^ Despite this, participants from both groups reported high levels of post-sleep alertness and good overall sleep quality. Interestingly, the average number of nightly awakenings were similar between the two groups. Our study did not investigate the specific causes of these awakenings; however, previous research provides some insights. In residential aged care, factors like environmental disturbances, for example, light and noise exposure, routine staff checks, and interruptions by other residents have been identified as common causes of sleep disruption alongside change in medications, cognitive status, the use of a walking aid, the average age of older adults and their health status.^11-13^ In contrast, older adults living in community settings often attribute their awakenings to personal health issues such as incontinence or the need to urinate, pain, change in medications and cognitive status, the use of a walking aid, and environmental noise within their homes.^11,13,18^

The current study found that there were no significant associations between sleep measures and fall occurrence, except for each minute subjectively reported awake, where the odds of experiencing a fall increased by approximately 1.8% (*P* = .037). The majority of the study population did not fall in the 9-month timeframe with the Australian Institute of Health and Welfare reporting those aged 65 years and older in April 2020 had 25% fewer hospitalizations that then same month in the previous year.^5^ However, to be practically meaningful, more studies would be required to confirm the direction of these associations. This is in contrast to previous research where associations found between sleep disturbances, sleep quality, sleep duration, and the risk of a fall.^14,42^ However, it is important to note there were some small significant associations found.

The data also revealed that for each additional awakening there was a significant decrease in falls risk, with an effect size (*R*² = .21) suggesting that 21% of the variance in falls risk could be attributed to number of awakenings for the simple task (*F*(1,29) = 7.75, β = −.69, *P* = .009, 95% CI [−1.20 to −0.18]) and cognitive task (*R*² = .13) (*F*(1,29) = 4.52, β = −.50, *P* = .042, 95% CI [−0.98 to −0.02]). These findings are contrary to the conventional understanding that uninterrupted sleep is most conducive to functional well-being in older adults. Typically, frequent awakenings are considered detrimental, potentially leading to daytime fatigue and impaired physical functioning.^14,19,42^ No other significant associations were found between sleep variables and falls risk. These results indicate that the relationship between sleep disturbances and physical functionality, especially in older adults requires further investigation.

This longitudinal observational study has a number of strengths. It employed a combination of subjective and objective sleep measures to investigate differences in measures of sleep between older adults in residential aged care and those living in the community. Furthermore, it examined the potential of these sleep parameters to predict falls and assess the overall falls risk. Unlike previous research, our study provided a more comprehensive longitudinal analysis of falls, gathering both objective and subjective data across multiple time points: baseline, 3 months, 6 months, and 9 months. By integrating this extensive falls data with sleep measurements taken at baseline, we aimed to better understand the predictive value of sleep patterns on the likelihood and risk of future falls. Additionally, building upon the limitations identified in another study,^38^ our research extended the duration of sleep diary entries from 3 to 7 days, enhancing the data’s depth and reliability.

A number of limitations which should also be considered. First, the absence of notable differences in sleep measures between older adults in residential aged care and those living in the community could stem from the limited sample size. Additionally, the lack of differences might be attributed to our sample comprising older adults who were recruited from exercise programs, who are generally healthier and more functional compared to those in previous studies. Consequently, a broader demographic, a larger, more diverse, sample size is needed in future studies. Second, sleep variables were only collected at baseline and not monitored over the follow-up periods of 3, 6, and 9 months. Future studies should collect both objective and subjective sleep data concurrently with falls data at multiple time points (eg, baseline, 3, 6, and 9 months) and then look to compare between walking ability. This approach will provide more robust and accurate results by capturing potential changes in sleep patterns over time.^21^ Third, the age difference between the residential aged care group and the community dwelling group could have influenced the results, as the former were generally older. This age disparity may have impacted findings related to health and fall status in regard to change in medications, cognitive status, hospitalization, and health status.^13^ Fourth, given the number of statistical analyses conducted, the potential of type 1 error warrants consideration. Post-hoc application of the false discovery rate^43^ resulted in some analyses no longer yielding statistically significant results, which reduces our confidence in our initial estimates. Finally, high attrition rates were observed during the study due to factors such as health deterioration, non-attendance, concerns of COVID-19 infection, and government-borne COVID-19 restrictions. Other factors, such as the fear of COVID-19 and restricted access to older adults also led to participant withdrawal. Future studies should consider strategies to mitigate attrition, such as flexible data collection methods and addressing participant concerns.

## Conclusion

There were no statistically significant differences in both subjective and objective measures of sleep when comparing sleep outcomes between residential aged care and community dwelling adults. Second, there were three notable findings when exploring subjective and objective sleep predictors for future falls and falls risk. For each minute spent awake (subjectively reported) increased the odds of experiencing a fall by 1.8%, for each additional awakening subjectively reported increased the odds of experiencing a fall by 65.3%, and a higher number of objectively measured awakenings during the night was consistently associated with a reduced risk of falls. Further studies will larger sample sizes are needed.

## Supplemental Material

sj-docx-1-inq-10.1177_00469580241306274 – Supplemental material for Sleep Health and Falls Risk for Older Adults Living in Residential Aged Care and in Community Dwelling Settings: A Longitudinal Observation Study Supplemental material, sj-docx-1-inq-10.1177_00469580241306274 for Sleep Health and Falls Risk for Older Adults Living in Residential Aged Care and in Community Dwelling Settings: A Longitudinal Observation Study by Samantha Fien, Natasha L. Bennett, Patrick Owen, Stephanie J. Alley, Corneel Vandelanotte, Madeline Sprajcer, Kim Waters, Steven T. Moore, Justin W.L. Keogh and Grace E. Vincent in INQUIRY: The Journal of Health Care Organization, Provision, and Financing

sj-docx-2-inq-10.1177_00469580241306274 – Supplemental material for Sleep Health and Falls Risk for Older Adults Living in Residential Aged Care and in Community Dwelling Settings: A Longitudinal Observation Study Supplemental material, sj-docx-2-inq-10.1177_00469580241306274 for Sleep Health and Falls Risk for Older Adults Living in Residential Aged Care and in Community Dwelling Settings: A Longitudinal Observation Study by Samantha Fien, Natasha L. Bennett, Patrick Owen, Stephanie J. Alley, Corneel Vandelanotte, Madeline Sprajcer, Kim Waters, Steven T. Moore, Justin W.L. Keogh and Grace E. Vincent in INQUIRY: The Journal of Health Care Organization, Provision, and Financing

sj-docx-3-inq-10.1177_00469580241306274 – Supplemental material for Sleep Health and Falls Risk for Older Adults Living in Residential Aged Care and in Community Dwelling Settings: A Longitudinal Observation Study Supplemental material, sj-docx-3-inq-10.1177_00469580241306274 for Sleep Health and Falls Risk for Older Adults Living in Residential Aged Care and in Community Dwelling Settings: A Longitudinal Observation Study by Samantha Fien, Natasha L. Bennett, Patrick Owen, Stephanie J. Alley, Corneel Vandelanotte, Madeline Sprajcer, Kim Waters, Steven T. Moore, Justin W.L. Keogh and Grace E. Vincent in INQUIRY: The Journal of Health Care Organization, Provision, and Financing
