# Supplementary material for: Sleep Health and Falls Risk for Older Adults Living in Residential Aged Care and in Community Dwelling Settings: A Longitudinal Observation Study
Source: Inquiry. 2024 Dec 17;61:00469580241306274. doi: 10.1177/00469580241306274 (PMC11650497; doi:10.1177/00469580241306274)
Supplement: sj-docx-1-inq-10.1177_00469580241306274 – Supplemental material for Sleep Health and Falls Risk for Older Adults Living in Residential Aged Care and in Community Dwelling Settings: A Longitudinal Observation Study [file sj-docx-1-inq-10.1177_00469580241306274.docx]

Supplementary File Two

*Objective sleep parameters and relevant definitions.*

| Sleep Parameter | Definition |
| --- | --- |
| Sleep latency | The time from the beginning of total recording time to the first epoch scored as sleep. |
| Sleep onset | Follows the latency period and is the first minute that the algorithm scores as sleep. |
| Total sleep time | The total amount of minutes scored as sleep. |
| Wake after sleep onset | The total amount of minutes the individual was awake after sleep onset had occurred. Wake after sleep onset is an indication of sleep fragmentation. |
| Number of awakenings | The number of times the participant awakened, as detected by the algorithm. |
| Average awakening length | Calculated in minutes, starting from the time the participant awakens. |
| Sleep efficiency | Calculated as the percentage of total time in bed, actually spent sleeping. |
| Movement index | The percentage of scored epochs with one or more activity counts during the sleep period, indicating movement during the sleep period. |
| Fragmentation index | The percentage of one minute periods of sleep vs. the count of all periods of sleep, during the time the individual spends sleeping. |
| Sleep fragmentation index | The sum percentage of the movement index and fragmentation index with higher values representing more fragmented sleep. |
