# Supplementary material for: Sleep Health and Falls Risk for Older Adults Living in Residential Aged Care and in Community Dwelling Settings: A Longitudinal Observation Study
Source: Inquiry. 2024 Dec 17;61:00469580241306274. doi: 10.1177/00469580241306274 (PMC11650497; doi:10.1177/00469580241306274)
Supplement: sj-docx-2-inq-10.1177_00469580241306274 – Supplemental material for Sleep Health and Falls Risk for Older Adults Living in Residential Aged Care and in Community Dwelling Settings: A Longitudinal Observation Study [file sj-docx-2-inq-10.1177_00469580241306274.docx]

Supplementary File Three

*Multiple regression model to predict a fall.*

| Time point | Predictor (objective) | Odds Ratio | Standard error | *z*-value | *p*-value | 95% confidence interval (upper – lower) |
| --- | --- | --- | --- | --- | --- | --- |
| **Falls (baseline)** | Total sleep time | 1.001 | 0.003 | 0.28 | 0.783 | 0.993 – 1.008 |
|  | Number awakenings | 0.990 | 0.064 | -0.14 | 0.887 | 0.871 – 1.008 |
|  | Average awakening length | 0.922 | 0.124 | -0.60 | 0.550 | 0.708 – 1.201 |
| **Falls**  **(3 months)** | Total sleep time | 1.201 | 0.014 | 1.44 | 0.150 | 0.992 – 1.048 |
|  | Number awakenings | 1.009 | 0.103 | 0.09 | 0.927 | 0.825 – 1.234 |
|  | Average awakening length | 0.464 | 0.339 | -1.05 | 0.295 | 0.111 – 1.947 |
| **Falls**  **(6 months)** | Total sleep time | 0.007 | – | – | – | – |
|  | Number awakenings | – | – | – | – | – |
|  | Average awakening length | – | – | – | – | – |
| **Falls**  **(9 months)** | Total sleep time | 0.996 | 0.005 | -0.66 | 0.507 | 0.984 – 1.007 |
|  | Number awakenings | 1.300 | 0.212 | 1.61 | 0.108 | 0.944 – 1.791 |
|  | Average awakening length | 2.160 | 0.900 | 1.85 | 0.064 | 0.954 – 4.819 |

*Note. There were not enough participants at the six-month time point. Odds ratio explains the odds of experiencing a fall, p value = statistical significance set at <0.05.*
